# Non-Volatile Memory and Synaptic Characteristics of TiN/CeO_x_/Pt RRAM Devices

**DOI:** 10.3390/ma15249087

**Published:** 2022-12-19

**Authors:** Hoesung Ha, Juyeong Pyo, Yunseok Lee, Sungjun Kim

**Affiliations:** Division of Electronics and Electrical Engineering, Dongguk University, Seoul 04620, Republic of Korea

**Keywords:** neuromorphic system, memristor, resistive switching, cerium oxide

## Abstract

In this study, we investigate the synaptic characteristics and the non-volatile memory characteristics of TiN/CeO_x_/Pt RRAM devices for a neuromorphic system. The thickness and chemical properties of the CeO_x_ are confirmed through TEM, EDS, and XPS analysis. A lot of oxygen vacancies (ions) in CeO_x_ film enhance resistive switching. The stable bipolar resistive switching characteristics, endurance cycling (>100 cycles), and non-volatile properties in the retention test (>10,000 s) are assessed through DC sweep. The filamentary switching model and Schottky emission-based conduction model are presented for TiN/CeO_x_/Pt RRAM devices in the LRS and HRS. The compliance current (1~5 mA) and reset stop voltage (−1.3~−2.2 V) are used in the set and reset processes, respectively, to implement multi-level cell (MLC) in DC sweep mode. Based on neural activity, a neuromorphic system is performed by electrical stimulation. Accordingly, the pulse responses achieve longer endurance cycling (>10,000 cycles), MLC (potentiation and depression), spike-timing dependent plasticity (STDP), and excitatory postsynaptic current (EPSC) to mimic synapse using TiN/CeO_x_/Pt RRAM devices.

## 1. Introduction

Recently, the amount of data and information have increased significantly with the development of new technologies such as artificial intelligence (AI) and the internet of thing (IoT) [1,2]. Therefore, data program/erase/read speed, power consumption, and operating voltage become significantly important. However, conventional memory devices such as flash memory have disadvantages in delaying memory access time because of their serial processing of information [3]. The human brain, which consists of approximately 10^12^ neurons and 10^15^ synapses, can compute and memorize data simultaneously in the same place [4,5]. Therefore, neuromorphic system devices, which mimic a biological system with parallel processing, are more suitable for energy-efficient and fast computing processing [6,7]. These devices are imitated by remembering, learning, and recognizing the functions of the human brain using neurons and synapses [8,9]. Resistive random-access memory (RRAM), which emulates the functionality of synaptic devices, has attracted enormous attention in the field of non-volatile memory (NVM) applications [10,11,12]. RRAM has several advantages, including long data retention, fast switching speed, long program/erase cycle endurance, and multi-level cell (MLC) capability [13]. 

The switching process of the RRAM is to change the resistance by applying an external voltage [14,15,16,17,18]. In other words, the RRAM stores “0” in the high resistance state (HRS) and “1” in the low resistance state (LRS). This resistive switching operation is possible because of the resistance change of the insulator in RRAM with bias. The sputtering system, which is a physical vapor deposition (PVD) technique for metal oxide deposition, permits several defects such as oxygen vacancies and grain boundaries [19,20,21]. Among them, the oxygen vacancies can be moved by an electrical force, providing a conducting path for electrons to flow [22]. There are major parameters in electrical signals such as voltage, time (width), and compliance current. By adjusting these parameters, the number of oxygen vacancies can be varied. The controlled vacancies exhibit multi-states rather than a simple “0” or “1” state [23]. As a synaptic device, the RRAM should represent the conductance for the weight of the synapse and should be implemented when the synapse (device) is stimulated (electrical signal). Excitatory postsynaptic current (EPSC) that is as conductance becomes fire indicates signal transmission in artificial neural networks [24,25]. In addition, continuously accumulated conductance is termed potentiation, and decreasing conductance is termed depression. Potentiation and depression are related to synaptic characteristics [26].

RRAM device consists of an insulating film sandwiched between two metal electrodes, which is called a metal-insulator-metal (MIM) structure [27]. The resistive switching properties are dependent on the material used for the metal electrode and the insulating layer. Various materials are used in insulator films, and transition metal oxides (TMO) are one of the most promising types. Because of its high compatibility, binary transition metal oxides, such as TaO_x_ [28], NiO [29], TiO _x_ [30], and HfO _x_ [31,32] have been studied. However, recent research results show that many rare-earth metal oxides, such as Gd_2_O_3_ [33], CeO_2_ [34], and Sm_2_O_3_ [35] also exhibit good resistive switching properties for NVM. Among them, the CeO _x_ thin films have attracted significant attention, owing to their strong ability to conduct oxygen ions or oxygen vacancy for RRAM devices [36,37]. The reaction of Pt and Al as top electrodes on the switching behavior of CeO_2_ films has also been studied [38,39]. In addition, the generation of oxygen vacancies was attributed to the effect between the CeO _x_ and TiN bottom layers when TiN was used as the bottom layer [40]. However, the resistive switching characteristics of CeO _x_ thin films still need to be studied systematically in various device configurations by using assorted top/bottom electrode materials.

This study reports on the effect of rare-earth transition metal oxides CeO_x_ films on synaptic properties and DC properties. The device is composed of a TiN material as the top electrode and Pt material as the bottom electrode. The top/bottom electrode materials influence the window and stability of the resistive switching. We report excellent bipolar switching properties in TiN/CeO_x_/Pt memory devices, which can provide a clear perception of the switching process and attractive options of dielectric materials for RRAM applications.

## 2. Experiments

The fabrication process of the TiN/CeO_x_/Pt device is as follows: a ~100-nm-thick Pt/Ti layer was formed as the bottom electrode on a SiO_2_/Si substrate wafer by a sputtering system. The Ti layer acts as an adhesion layer between Pt and SiO_2_, regardless of the electrical characteristics. After that, a ~20 nm CeO_x_ switching layer was deposited by a reactive sputtering system using a 3-inch Ce target. The Ar flow rate was 20 sccm, the O_2_ flow rate was 6 sccm, and the working pressure was 5 mTorr during sputtering at room temperature (RT). Finally, to deposit the top electrode with a thickness of 100 nm, a Ti metal target was reactive sputtered in an ambiance of N_2_ and Ar gases. The photolithography and lift-off process was performed to separate the cells from each other when depositing the top electrode. The square cell area is 100 μm × 100 μm. To analyze the electrical characteristics, Keithley’s 4200-SCS and 4225-PMU semiconductor parameter modules were utilized for DC sweep and pulse switching, respectively. The ground was applied to Pt BE while voltage bias was applied to TiN TE.

## 3. Results and Discussions

**Figure 1:**
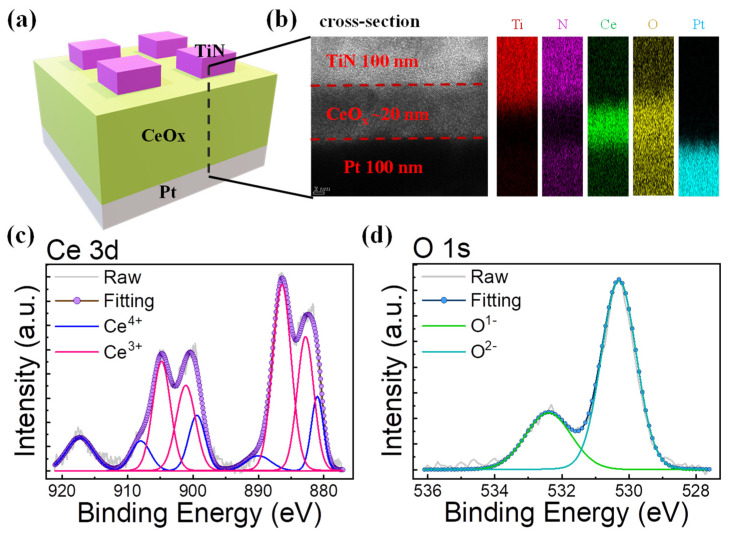
(**a**) Schematic of TiN/CeO_x_/Pt RRAM cells, (**b**) cross-sectional TEM image with EDS mapping that consists of Ti (red), N (purple), Ce (green), O (yellow), Pt (sky blue), and (**c**) Ce 3d and (d) O 1s XPS spectra of CeO_x_ film.

Figure 1a shows the schematic of the fabricated RRAM device. The TiN top electrode on the CeO_x_ layer is separated at regular intervals to operate resistive switching in a cell of 100 × 100 μm area. Figure 1b shows the transmission electron microscopy (TEM) image and the energy dispersive spectrometer (EDS) mapping of the TiN/CeO_x_/Pt stack. The thickness of the CeO_x_ layer is approximately 20 nm. Figure 1c,d shows the X-ray photoelectron spectroscopy (XPS) binding energy profiles of Ce 3d and O 1s for CeO_x_ insulator film. Figure 1c shows the Ce^3+^ and Ce^4+^ spectra in the XPS spectra of Ce 3d. This indicates that a non-stoichiometric state CeO_x_ film was deposited (1.3 < x < 2) [38]. Figure 1d shows the O 1s spectrum. The O 1s spectrum can be fitted by two peaks, corresponding to the binding energies of approximately 528.5 and 530.7 eV. In the binding energies of the two peaks, the higher energy (green fitting line) is assigned to O^1−^ ions, and the other binding energy (sky blue fitting line) is assigned to O^2−^ ions. The O^1−^ ions and O^2−^ ions denote the non-lattice oxygen and lattice oxygen, respectively [41,42]. The non-lattice oxygen ions contribute to resistive switching. In addition, the atomic concentration is calculated, and the result shows that the ratio of O to Ce is approximately 1.69 in CeO_x_ bulk.

**Figure 2:**
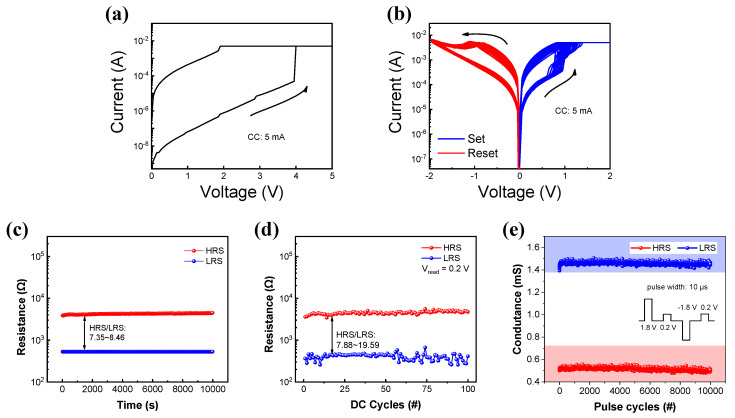
I-V curves of (**a**) forming process and (**b**) bipolar resistive switching including set and reset processes. (**c**) Retention and (**d**) endurance test by DC sweep. (**e**) Endurance test by pulse mode.

Figure 2a shows the I-V curve of the forming process of the device. Initially, an extremely high current flows, indicating that the CeO_x_ layer has many oxygen vacancies. Figure 2b shows the repetitive I-V curves of the TiN/CeO_x_/Pt device. The set and reset process occurs repetitively by the positive bias and negative bias, respectively. The current begins to increase noticeably between 0.5 V and 1 V and is limited by a compliance current of 3 mA. The set process changes the device state from a high-resistance state (HRS) to a low-resistance state (LRS) by increasing the conducting defects in the CeO_x_ layer. The oxygen vacancies (ions) near the interface of the CeO_x_ and Pt (TiN) layer increase during the set process [43]. At positive bias voltage, the current in the backward sweep is higher than the current in the forward sweep for resistive memory switching. On the contrary, the reset process occurs when a negative bias is applied to the device. A gradual resistance change is observed during the reset process. The resistance in the RRAM device increases by the recombination between the oxygen and oxygen vacancies. In addition, the set voltage is significantly lowered in the paper reported Pt/CeO_x_/Pt [44] with only different top electrodes. This phenomenon allows the TiN to absorb oxygen ions and can operate the device as LRS at low voltage. Non-volatile memory properties in the LRS and HRS are verified by a retention test. HRS and LRS are nearly constant without degradation for 10,000 s, as shown in Figure 2c. Moreover, Figure 2d,e shows the endurance cycle test by DC sweep and pulse mode, respectively. The LRS and HRS at the reading of 0.2 V have enough on/off ratio (>10) for 100 cycles in DC mode. In pulse mode, the conductance ratio (LRS and HRS) exhibits identical stability.

**Figure 3:**
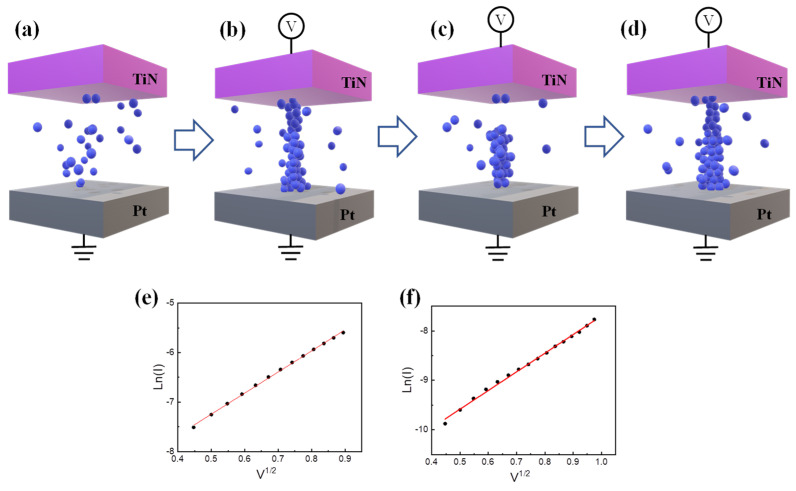
(**a**–**c**) Schematic diagram of the filament evolution for (**a**) initial state, (**b**) forming, (**c**) reset, and (**d**) set. ln(I) versus V^1/2^ for Schottky emission of (**e**) LRS and (**f**) HRS.

Figure 3a–d shows the schematics of resistive switching based on the filamentary model for the TiN/CeO_x_/Pt device. The CeO_x_ layer has many oxygen vacancies in the initial state owing to the reactive sputtering (PVD), as shown in Figure 3a. The oxygen vacancies form the conducting path at a specific threshold voltage. It is usually known as the electroforming process or soft breakdown in Figure 3b. Subsequently, the filament is partially ruptured by the reset process, as shown in Figure 3c. At this time, the oxygen ions (anions) and the oxygen vacancies (cations) are electrically bonded [45]. Conversely, in Figure 3d, the filament gap is refilled by the set process again. The gap could be related to the Schottky barrier, which is verified by the fitting of ln(I) versus V^1/2^ in Figure 3e,f [39].

**Figure 4:**
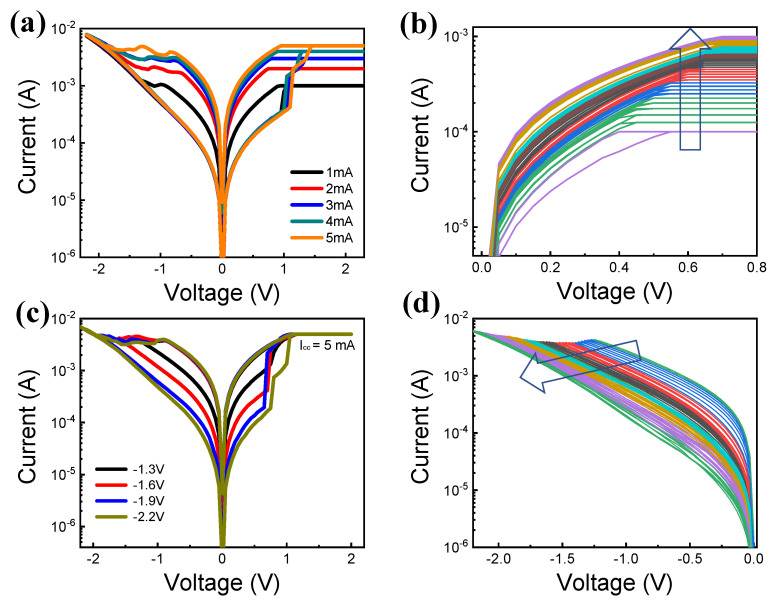
(**a**) I-V curves by controlling compliance current (1 mA, 2 mA, 3 mA, 4 mA, and 5 mA) and (**b**) MLC set process by increasing compliance current. (**c**) I-V curves by reset stop voltage (−1.3 V, −1.6 V, −1.9 V, and −2.2 V) and (**d**) MLC reset process by fine control of reset voltage.

Next, we demonstrate the MLC by controlling the compliance current and resetting the stop voltage in the DC sweep. Figure 4a shows the I-V characteristics with different compliance current conditions (1 mA, 2 mA, 3 mA, 4 mA, and 5 mA) and the same reset stop voltage of −2 V. As the compliance current increases, the LRS current increases. However, the HRS current always returns to an almost constant value by the same reset stop voltage. Moreover, the fine control of the resistance state is achieved by compliance current, as shown in Figure 4b. The compliance current is varied from 0.1 mA to 1 mA in increments of 0.025 mA. As the compliance current increases, it is confirmed that the current generally increases. Figure 4c shows the I-V characteristics by controlling the reset stop voltage (−1.3 V, −1.6 V, −1.9 V, and −2.2 V) with the fixed compliance current (5 mA). The current decreases on increasing the absolute voltage of the reset stop voltage. However, the LRS current is maintained constant by controlling the same compliance current. Moreover, we demonstrate the clear adjustment of the current by changing the reset stop voltage from −1.25 V to −2.2 V in increments of −0.025 V. In this case, a more gradual reset transition is observed as compared to the set transition.

**Figure 5:**
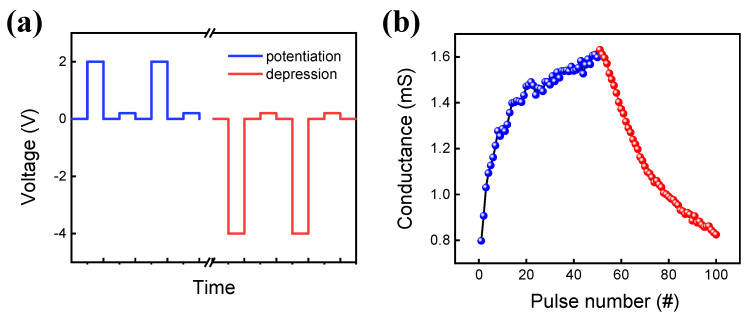
(**a**) Pulse schematic. (**b**) Potentiation and depression in cycles.

Next, we study the conductance control by a pulse for a more practical measurement system. Firstly, the pulse endurance is performed by a set pulse of 1.8 V and a reset pulse of −1.8 V with a pulse width of 10 μs, as shown in Figure 5a. The voltage of the read pulse is 0.2 V to minimize the read disturbance on the RRAM device. The long switching (10,000 cycles) in the pulse mode [46] can be endured although the on/off ratio is smaller than that of DC endurance. Figure 5b shows the potentiation and depression curves that are essential to implement the hardware-based neuromorphic system [47]. The set pulse (amplitude voltage: 1.8 V, width: 100 μs) and reset pulse (amplitude voltage: −1.8 V, width: 100 μs) are used for potentiation and depression, respectively.

Finally, we emulate the spike-timing dependent plasticity (STDP) and excitatory postsynaptic current (EPSC) of biological synapses using the gradual resistive switching characteristics of the RRAM device. STDP is a biological process that controls the strength of the connections between neurons via synapses in the brain. The basic rule of STDP is based on the relative timing of a particular neuron’s input spike and output spike.

**Figure 6:**
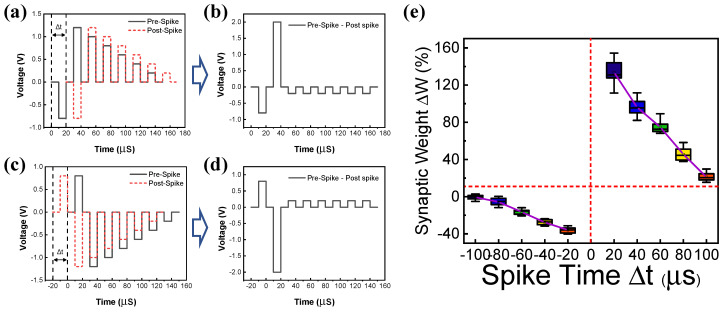
(**a**) Pulse schemes of pre-spike (black line) and post-spike (red line). (**b**) Pre-spike occurs before post-spike by Δt, causing the overlapped pulse for potentiation. (**c**) Pulse schemes of pre-spike (black line) and post-spike (red line). (**d**) Pre-spike occurs after post-spike by Δt, causing the overlapped pulse for depression. (**e**) Synaptic weight change as a function of spike time for STDP emulation.

**Figure 7:**
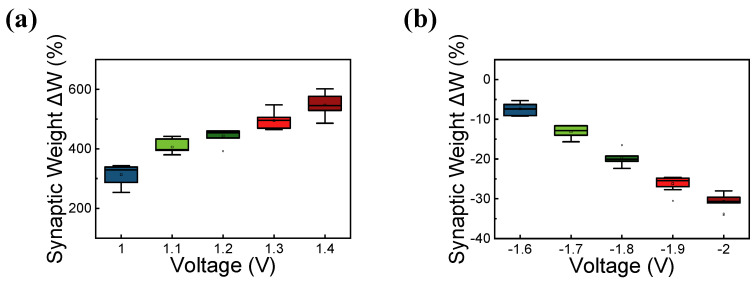
EPSC characteristics in (**a**) positive voltage and (**b**) negative voltage.

As the time interval decreases, the synaptic weight increases. EPSC is a postsynaptic potential that induces the postsynaptic neuron to easily fire an action potential [48]. Figure 6a,b shows the overlap of pulse trains for potentiation in which the effective set pulse is applied to the device. The overlapped pulse indicates that a pre-spike appears before the post-spike by Δt. Similarly, Figure 6c,d shows the overlap of pulse trains that a pre-spike appears after post-spike for depression. A negative voltage is effectively applied to the devices. Figure 6e shows the STDP characteristics by applying a pulse train consisting of five pulses on the device. Spike-timing-dependent synaptic weight is well emulated in the RRAM device. Figure 7a,b shows the change in the synaptic weight as a function of pulse voltage for EPSC. The change in the synaptic weight increases with increasing the absolute value of the voltage for both positive and negative voltage regimes.

## 4. Conclusions

The resistive switching characteristics of TiN/CeO_x_/Pt RRAM devices are investigated for non-volatile memory and neuromorphic system applications. The I-V curves are obtained by DC sweep for bipolar resistive switching. Moreover, 100 cycles of repetitive DC endurance and stable retention for 10,000 s are demonstrated to prove the reliability of the RRAM as a memory device. The filamentary switching with gap and Schottky emission-based conduction models that indicate resistive switching possibility are also presented. The compliance current and reset stop voltage to implement MLC could control the gradual resistance change in the set and reset processes. Compared to the set transition, a more gradual reset transition is exhibited. Furthermore, stable longer pulse endurance and MLC are obtained not only in DC mode but also in pulse mode by set and reset pulses. Finally, STDP and EPSC in biological systems are mimicked using TiN/CeO_x_/Pt RRAM devices for neuromorphic systems. The larger change in the synaptic weight is observed as the interval time between the pair stimuli is shorter or the stimuli are stronger.

## Data Availability

Not applicable.
